# 

**DOI:** 10.1017/psy.2025.10030

**Published:** 2025-09-02

**Authors:** Muwon Kwon, Nathan Quimpo, Peter Steiner

**Affiliations:** Department of Human Development and Quantitative Methodology, University of Maryland, College Park, MD, USA

Recent advances in machine learning (ML) and artificial intelligence (AI) methods have given social scientists the tools to analyze large, high-dimensional, and multimodal datasets (including text, image, and video data). ML offers procedures to generate predictive models in high dimensions, where the number of predictors is large relative to the number of observations. AI enriches ML methods by using feature engineering to extract numerical features from complex nonnumerical data, such as open-ended survey responses, interview transcripts, or images. However, ML/AI-based methods have traditionally focused on *prediction* while ignoring *statistical* and *causal inference*, despite the fact that many questions in the social sciences require both statistical inference about correlational effects (i.e., generalizing an *observed* correlational effect of a predictor on an outcome from a sample to a population) and causal inference (i.e., claims about a causal effect if one were to *intervene* on a predictor). The past few years have seen significant advances in integrating ML/AI-based methods for predictive and causal inference, and Chernozhukov et al.’s (2025) recent textbook, *Applied Causal Inference Powered by ML and AI*, provides an accessible and practice-oriented introduction to an otherwise advanced and highly technical body of literature (currently, it is available only as a downloadable online textbook: updated as of March 5, 2025).

The book’s Core Material, which comprises the first 10 chapters, is organized toward building an understanding of the essential concepts behind the textbook’s main method, double/debiased machine learning (DML). DML is a statistical method designed to estimate predictive (i.e., correlational) or causal effects and their standard errors while controlling for a potentially high-dimensional set of covariates using machine learning. The chapters of the Core Material are organized into three central concepts—“Prediction,” “Inference,” and “Causality”—which are explored in an interleaved manner to build a cohesive understanding. Accordingly, this review is structured by concept rather than by the book’s chapter sequence. The interweaving of these different subjects reflects the book’s two central themes. First, high-quality prediction enriches both predictive and causal inference by deriving more precise estimates of the parameters of interest. Second, what distinguishes predictive and causal inference is often not the specific statistical method that is applied, but whether the researcher imposes plausible causal assumptions on the data, such as unconfoundedness. AI methods such as feature engineering complement both themes by transforming complex data into a tractable collection of numerical features. This process provides a greater number of potential control variables that can be used to achieve greater prediction accuracy, more precise predictive and causal inference, and enhanced control for confounders, thereby strengthening the credibility of causal inference. The book also includes seven additional chapters on advanced topics, which cover methods for assessing effect heterogeneity as well as standard identification strategies for causal inference, such as instrumental variables, difference-in-differences, and regression discontinuity designs. Throughout, the emphasis is on how ML and DML techniques can be applied to identify and estimate causal effects.

The chapters related to “Inference” and “Prediction” lay the foundation for later discussions of “Causality” and its associated assumptions and frameworks. Chapter 1 sets the stage by introducing linear regression for prediction and its statistical properties when making predictive inferences in low-dimensional settings. Importantly, the authors frame regression as a flexible method for approximating the conditional expectation function. They dispense with some of regression’s traditional assumptions such as linearity (by using flexible transformations of the regressors that can approximate nonlinear functional forms), heteroscedasticity (by using the Eicker–Huber–White robust variance estimator), and normality—they only maintain the requirement for independent and identically distributed data. Given an outcome variable, the chapter focuses on estimating the average predictive effect (APE) of a single focal predictor (e.g., a treatment variable) by using the partialling-out procedure. This procedure removes the influence of control variables from both the focal predictor and the outcome, and then estimates the APE by regressing the residualized outcome on the residualized focal predictor (Frisch & Waugh, 1933; Lovell, 1963, 2008). Then, the authors highlight why traditional predictive inference breaks down as the dimensionality of the potential predictors increases considerably, particularly as flexible functional forms need to be considered. Chapter 3 extends linear regression to high-dimensional settings through the least absolute shrinkage and selection operator (Lasso) for covariate selection to reduce the dimensionality of the predictors (while also introducing other regularization methods such as ridge regression, elastic nets, and lava). Although Lasso allows for prediction in high-dimensional settings, it also introduces regularization bias as it shrinks all coefficients toward zero, resulting in an underestimation of large coefficients. To address this regularization bias, Chapter 4 introduces the double Lasso for estimating the APE. In this approach, Lasso is used twice to residualize both the focal predictor and the outcome by partialling out the effects of potentially high-dimensional covariates. The authors then introduce Neyman orthogonality, a key property of the double Lasso estimator of the predictive effect, under which the estimator is locally insensitive to perturbations of the nuisance functions and therefore to regularization bias. Finally, Chapter 8 introduces different ML methods using nonlinear regression such as regression trees, random forests, boosted trees, (deep) neural networks, and ensemble methods, which offer the potential for more accurate prediction at the cost of drawing statistical inferences of interpretable parameters.

After establishing the foundations for predictive inference, the chapters on “Causality” build on this groundwork by examining the connections between predictive and causal inference. In particular, they highlight the additional assumptions that are required to interpret a predictive effect causally. Chapter 2 introduces causal inference with randomized experiments using the potential outcomes framework for defining average causal effects (ACEs). The key assumptions to identify causal effects are introduced, such as consistency, absence of interference, and exogeneity. They also introduce selection bias as an explanation for why the APE can differ from the ACE. Chapter 5 extends this discussion beyond randomized experiments into situations where confounding variables influence both the treatment and the outcome, which introduces selection bias. It introduces the conditional ignorability assumption, which requires identifying a set of covariates that successfully removes all the selection bias, and statistical methods to estimate the causal effect using both regression and inverse probability weighting based on the propensity score. Chapters 6 and 7 introduce linear and nonlinear SEMs (also known as DAGs) as a method for formalizing the causal assumptions that allow us to systematically determine the covariate set that satisfies the conditional ignorability assumption.

Finally, Chapter 9 introduces DML, which draws on all of the conceptual tools developed earlier. Building on the discussion of the double Lasso in Chapter 4, this chapter presents the general DML framework for estimating average predictive and causal effects, along with their standard errors. This chapter discusses the two key strategies of DML: the partialling-out approach and sample-splitting. Partialling-out enables the DML estimators to achieve Neyman orthogonality, thereby eliminating regularization bias. Sample-splitting is essential for guarding against overfitting bias that arises when the same data is used to estimate both nuisance parameters and the target parameter. By partitioning the sample into independent subsets—one for estimating nuisance parameters and another for estimating the target parameter—sample-splitting breaks the dependence between the two estimations, thereby removing overfitting bias. Chapter 10 introduces AI methods for feature engineering, including autoencoders, neural networks, and natural language processing algorithms like Embeddings from Language Models (ELMo) and Bidirectional Encoder Representations from Transformers (BERT). These methods transform complex data such as texts and images into a collection of numerical features, which are then used as additional predictors for predictive and causal inferences.

One of the book’s greatest strengths is its introduction to modern ML and AI methods with a balance of both rigor and accessibility. This book covers a wide range of methods, such as linear regression; penalized regression methods such as Lasso, ridge regression, and elastic net; tree-based methods such as regression trees, random forests, and boosted trees; (deep) neural network models; and DML. In addition to motivating each of these methods, the authors also discuss the assumptions and necessary conditions for accurate and efficient prediction performance, as well as their limitations. For example, when introducing penalized regression methods in Chapter 3, the authors identify the different scenarios in which each method performs best, such as when the coefficients are approximately sparse, dense, or a mix of both. The textbook also describes the key theorems about the estimators’ convergence to the population parameter and the variability of the estimates around the population parameter for standard error estimation. Furthermore, the authors put significant effort into explaining the choice of the tuning parameter for Lasso, which is often simply treated as the result of cross-validation. They provide a theoretical rationale for selecting a valid tuning parameter, which ensures good predictive performance of Lasso. Additionally, to demonstrate some key concepts, the authors provide relevant Python and R Notebooks. Each Notebook begins with a clear explanation of the purpose of the code implementation, ensuring that readers understand the objectives before running the analyses. The scripts are also well-structured and easy to follow, making them accessible even to those with limited programming experience, and are directly connected to Google Colab, allowing readers to run and interact with the code seamlessly.

Another excellent aspect of this book is the authors’ use of causal graphs in every chapter, which primarily focuses on causal inference. One of the main advantages of causal graphs is that they make explicit most assumptions required for identifying causal effects (Pearl, 2009; Spirtes et al., 2000; Steiner et al., 2017). Given that DML for causal inference relies on the unconfoundedness assumption—requiring a set of observed variables that blocks all noncausal paths simultaneously—causal graphs play a crucial role in covariate selection within the DML framework. To provide this benefit to readers, even to those unfamiliar with causal graphs, the authors consistently present causal graphs throughout Chapters 2, 5, and 6 with clear explanations, and then link them to the corresponding (nonparametric) structural causal models in Chapter 7. Although causal graphs are powerful tools that automatically identify a covariate set that satisfies the unconfoundedness assumption, one of the biggest challenges of using causal graphs is the process of constructing the graph itself (Imbens, 2020). The connections between causal graphs and applications could have been better illustrated when discussing the applied examples by showing how to construct a causal graph and demonstrating how it should be used to select an initial, potentially high-dimensional covariate set that meets the unconfoundedness assumption.

In summary, this book gradually builds readers’ understanding of DML methods by introducing relevant concepts step by step in a coherent manner. The lengths of the chapters are typically controlled between 20 and 40 pages, which is appropriate for weekly reading. These features of the book align well not only with its target audience—upper-level undergraduates and graduate students—but also with applied researchers, all of whom are expected to have at least one semester of experience in introductory ML, causal inference, and statistics, including basic regression and probability theory. Furthermore, these features make the book highly suited for teaching, as they provide instructors with a clear framework for course design (including Python and R Notebooks) and allow for manageable reading assignments that support a structured and effective learning. Depending on students’ prior exposure to ML or causal inference, a one-semester course might cover the foundations of only the first 10 chapters (Core Material) or include the entire book, including the Advanced Topics, particularly if the course focuses on causal inference. We highly recommend this book to instructors and students in the social and behavioral sciences, as it successfully introduces the new, technical language used in computer science, statistics, and econometrics to discuss regression-based predictive and causal inference in the context of AI and ML.
